# Supplementary material for: Palladium(II) and Platinum(II) Complexes Bearing ONS-Type Pincer Ligands: Synthesis, Characterization and Catalytic Investigations
Source: Molecules. 2024 Jul 22;29(14):3425. doi: 10.3390/molecules29143425 (PMC11280083; doi:10.3390/molecules29143425)
Supplement: Supplementary file 1 [file molecules-29-03425-s001.zip › molecules-3114097-supplementary.pdf]

Molecules

Palladium(II) and Platinum(II) complexes bearing ONS-type pincer ligands. Synthesis, characterization and catalytic investigations.

Elena Bermejo, Alfonso Castiñeiras, Isabel García-Santos

Universidad de Santiago de Compostela, Departamento de Química Inorgánica,

Facultad de Farmacia, Santiago de Compostela, 15782, Spain

Supplementary material

Table S1. Hydrogen bond parameters pag. 2

Table S2. Intermolecular ring-ring interaction parameters pag. 2

Figure S1. Mass spectra pag. 3

Figure S2. FT-IR spectra in the region 4000-500 cm^{-1} pag. 4

Figure S3. FT-IR spectra in the region 500-100 cm^{-1} pag. 6

Figure S4. UV-visible Spectra pag. 7

Figure S5. ^{1}H NMR Spectra pag. 8

Figure S6. ^{13}C NMR Spectra psg. 12

Figure S7. ^{195}Pt NMR Spectra pag. 14

Table S1. Hydrogen bond parameters [Å, °]

Compound	D–H···A	D–H	H···A	D···A	∠DHA	Symmetry
**3**	N(14)-H(14A)···Cl(1)[a]	0.77(5)	2.80(5	3.452(4)	144(5)	x,-y+1/2,z+1/2
**5**	N(14)-H(14A)···Cl(1)[a]	0.76(4)	2.75(4)	3.500(3)	171(4)	x-1/2,-y-1/2,z-1/2

Table S2. Intermolecular π···π interaction parameters (Å, º)

Compound	π···π	Cg(I)···Cg(J)	α
**3**	Cg(1)···Cg(3)[f]	3.608(2)	13.38(17)
	Cg(1) ring (Pd1/S1/C17/N13/N12)		
	Cg(3) ring (N11/C11/C12/C13/C14/C15)		
	f = 1-*x*, -*y*, -*z*		
**5**	Cg(1)···Cg(3)[b]	3.701(2)	13.27(15)
	Cg(3)···Cg(4)[c]	3.623(2)	8.56(19)
	Cg(1) ring (Pd1/S1/C17/N13/N12)		
	Cg(3) ring (N11/C11/C12/C13/C14/C22)		
	Cg(5) ring (C14/C15/C16/C17/C21/C22)		
	b = -*x*,-*y*,-*z*		
	c = -*x*+1,-*y*,-*z*		

Cg(I)···Cg(J): Distance between ring centroids; α: Dihedral angle between planes I and J. For details, see Janiak, C. A critical account on π–π stacking in metal complexes with aromatic nitrogen-containing ligands. *J. Chem. Soc. Dalton Trans.* **2000**, 3885–3898.

Figure S1. Mass spectra of the complexes (FAB).

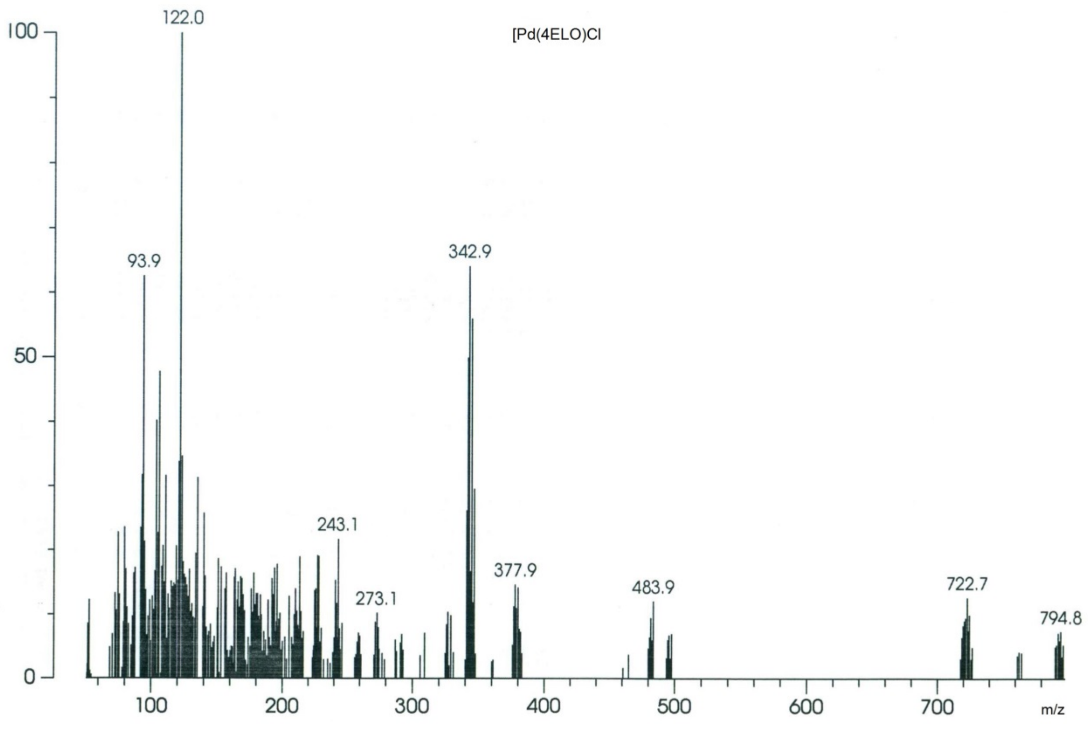

[Pd(4ELO)Cl] (**3**)

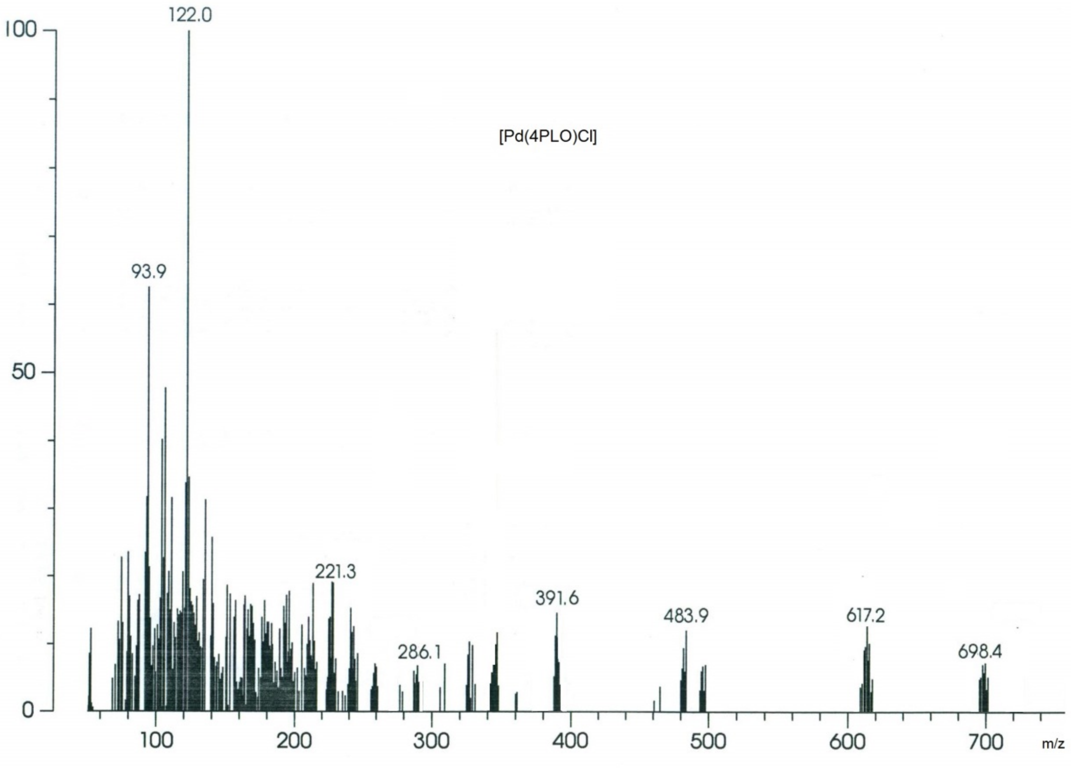

[Pd(4PLO)Cl)(**5**)

Figure S2. FT-IR spectra in the region 4000-500 cm^{-1}

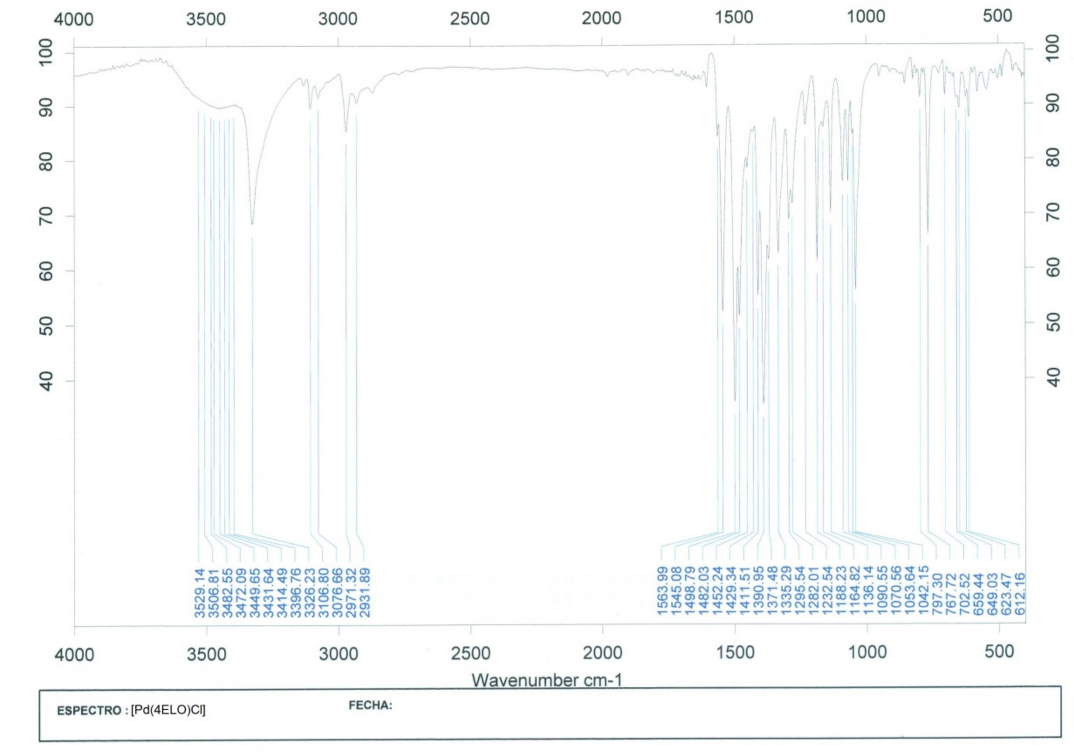

[Pd(4ELO)Cl](**3**)

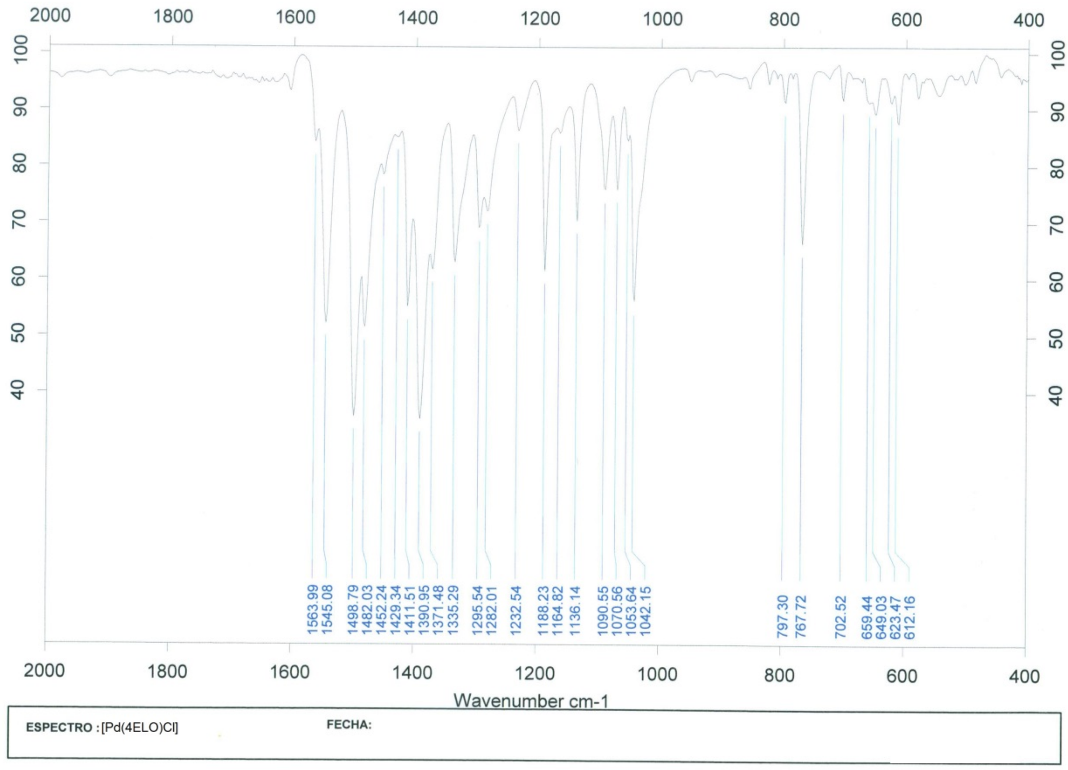

[Pd(4ELO)Cl (**3**)

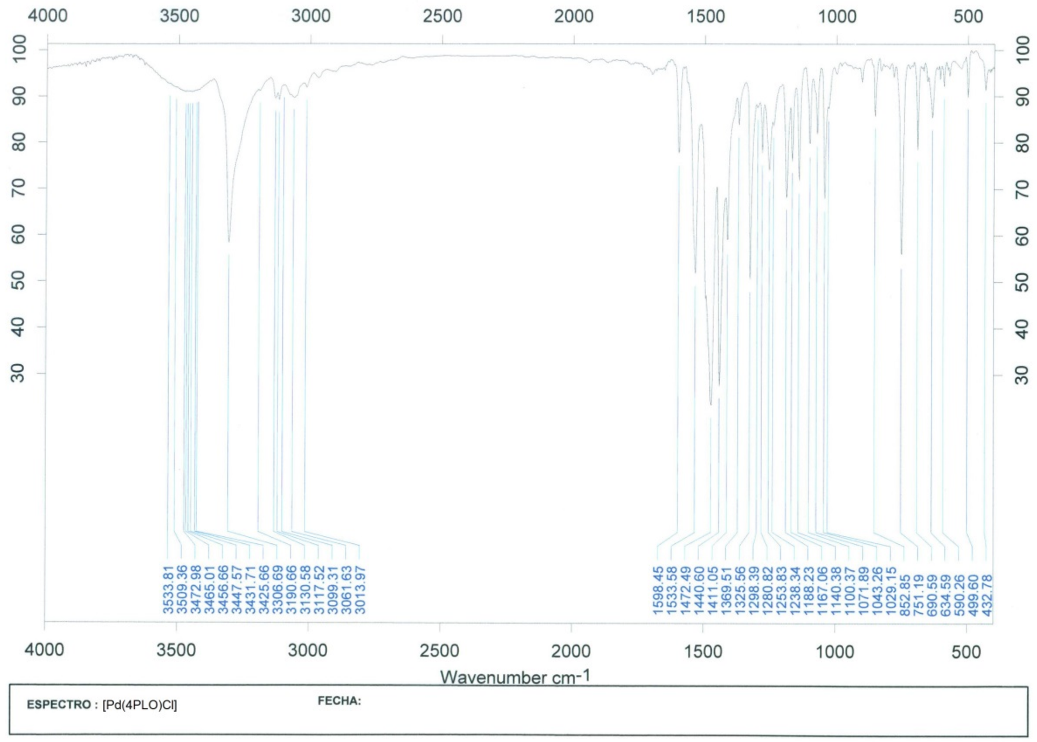

[Pd(4PLO)Cl] (**5**)

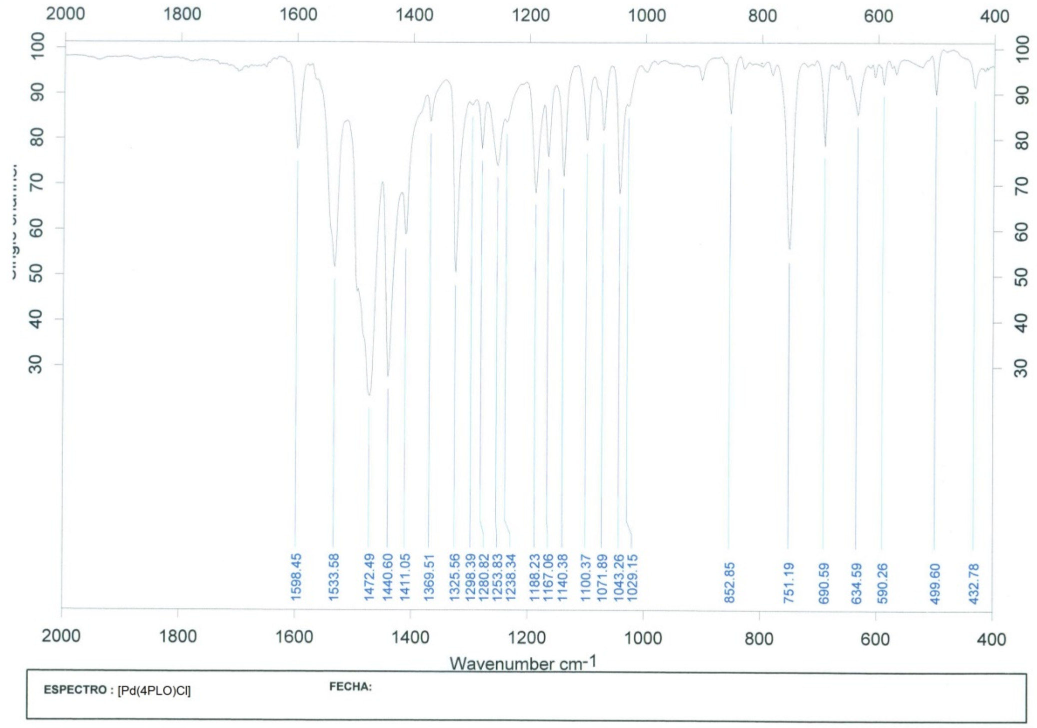

[Pd(4PLO)Cl] (**5**)

Figure S3. FT-IR spectra in the region 500-100 cm^{-1}

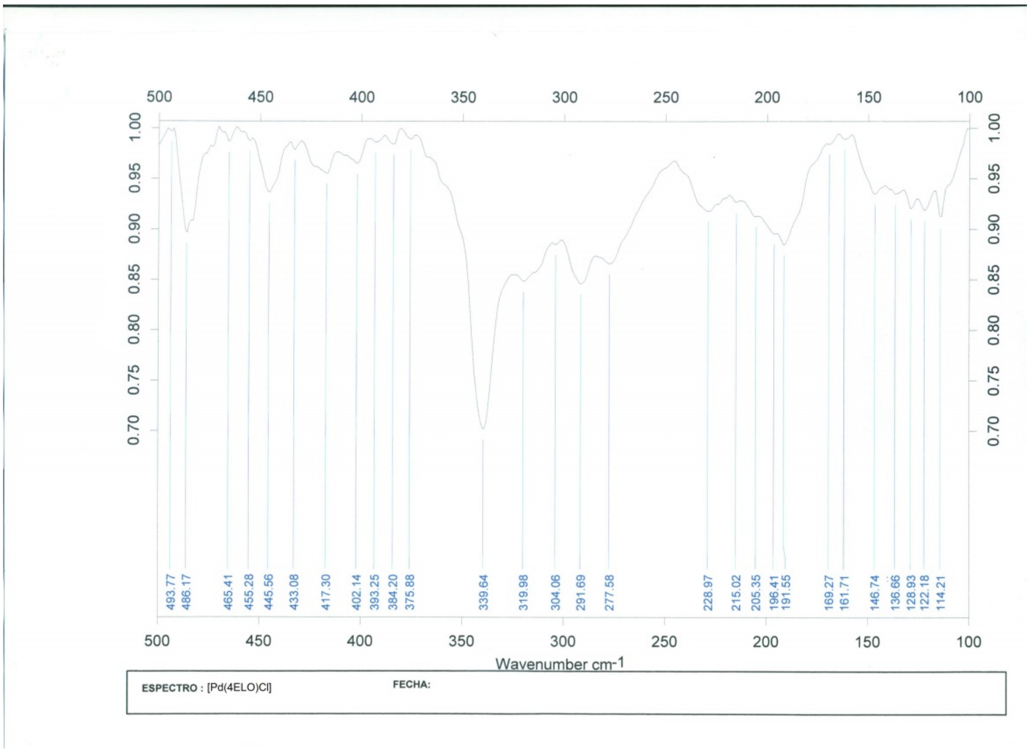

[Pd(4ELO)Cl] (**3**)

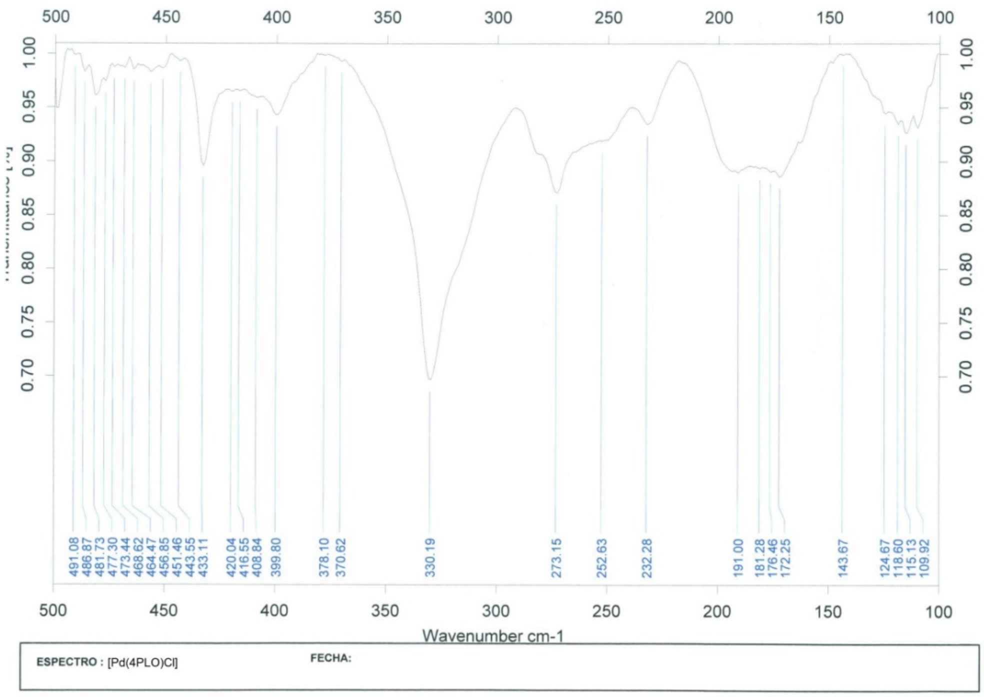

[Pd(4PLO)Cl] (**5**)

Figure S4. UV-visible Spectra

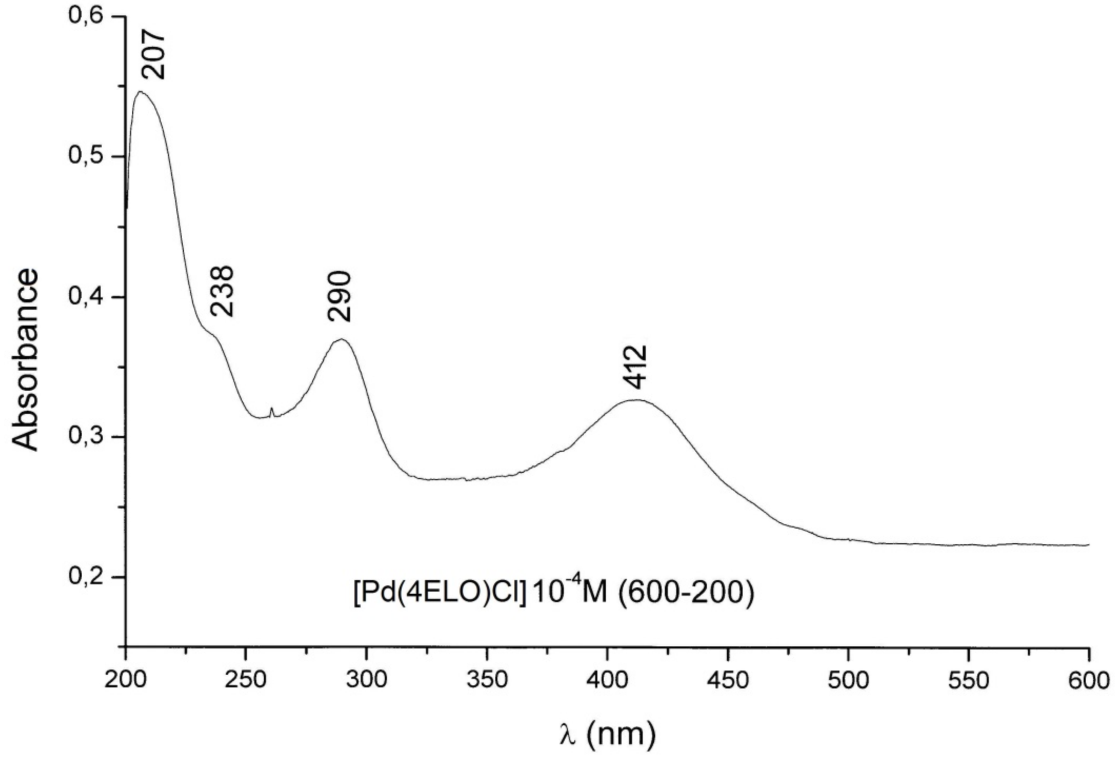

[Pd(4ELO)Cl] (**3**)

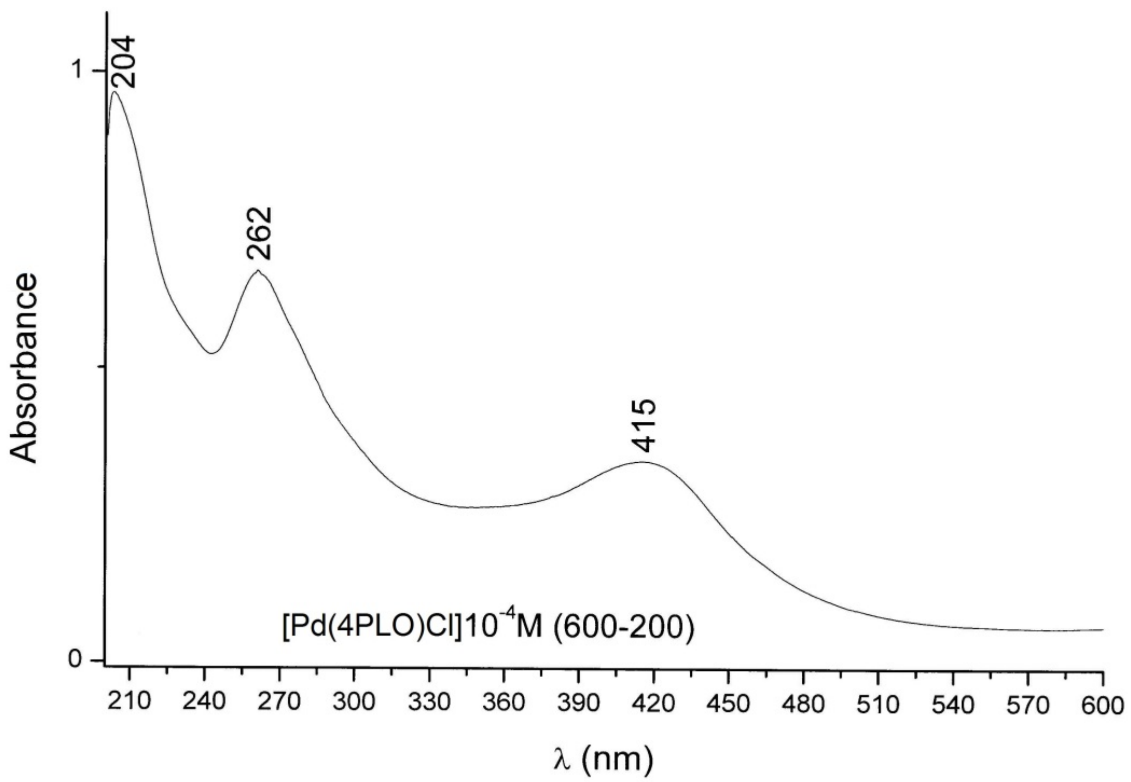

[Pd(4PLO)Cl] (**5**)

Figure S5. ^{1}H NMR spectra

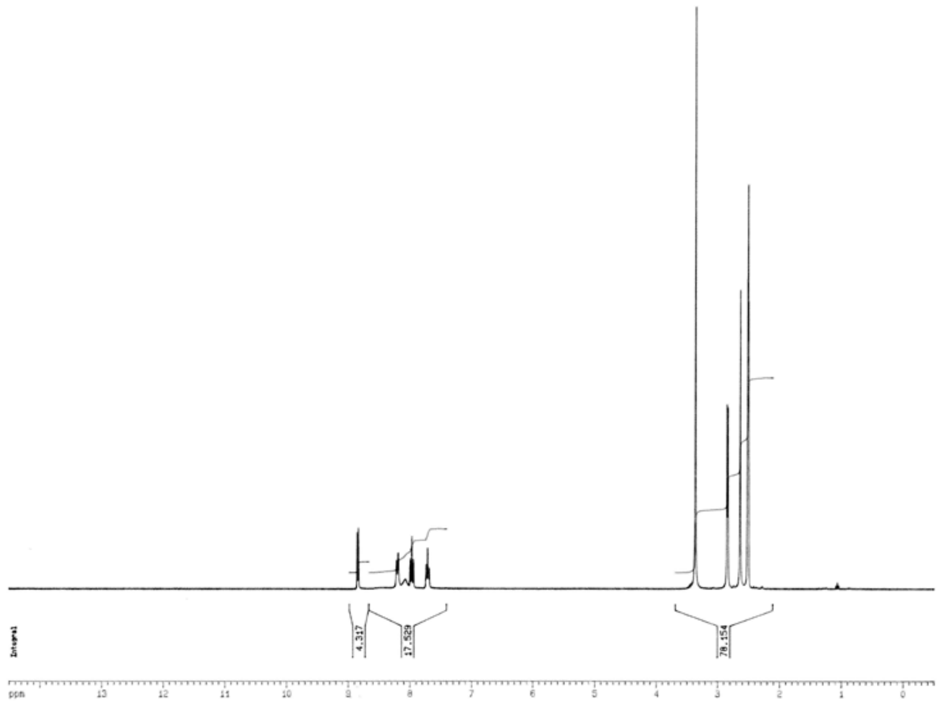

[Pd(4MLO)Cl] (**1**)

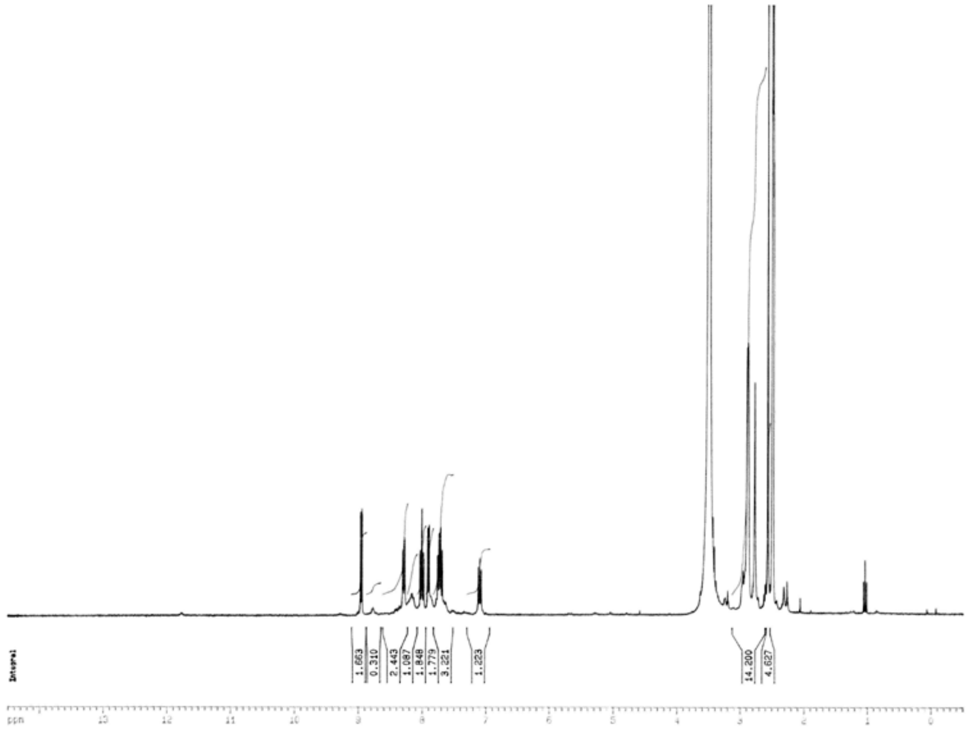

[Pt(4MLO)Cl] (**2**)

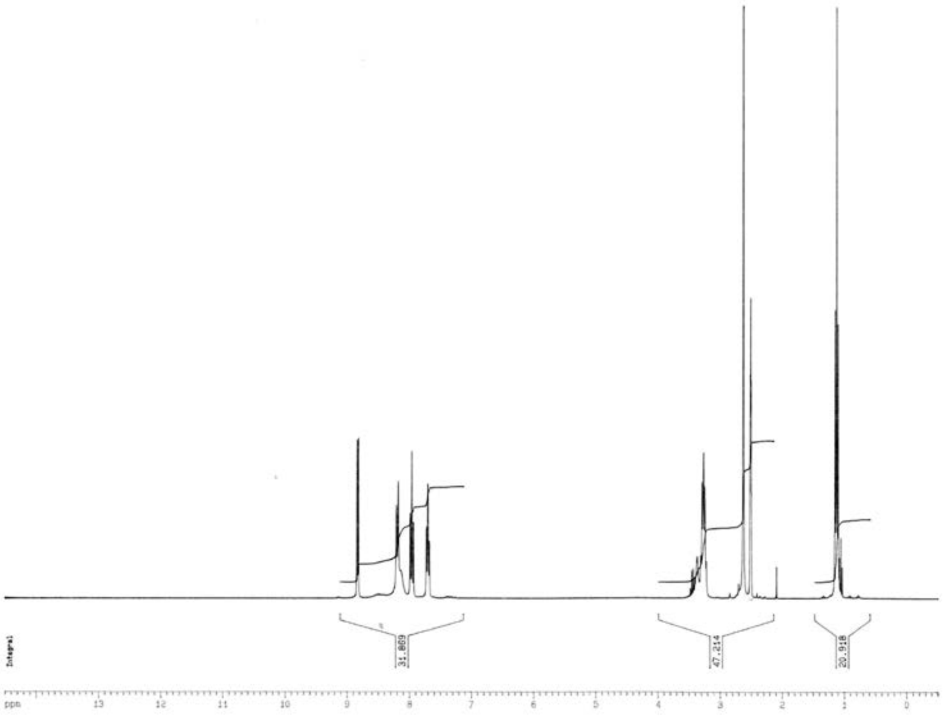

[Pd(4ELO)Cl] (**3**)

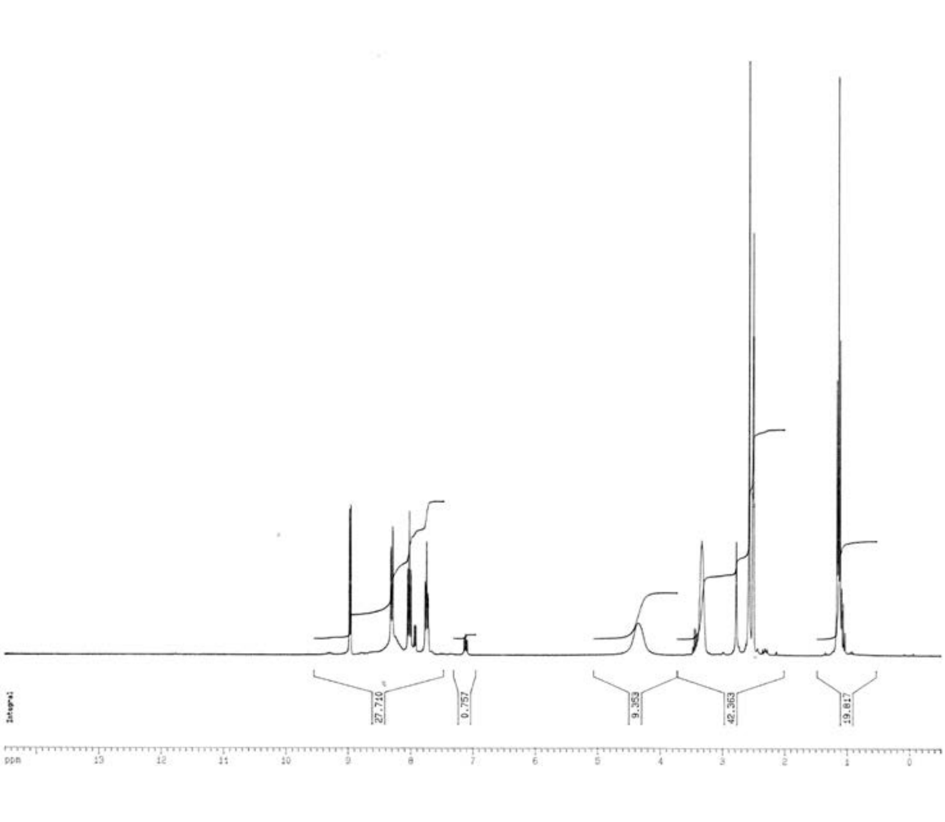

[Pt(4ELO)Cl (**4**)

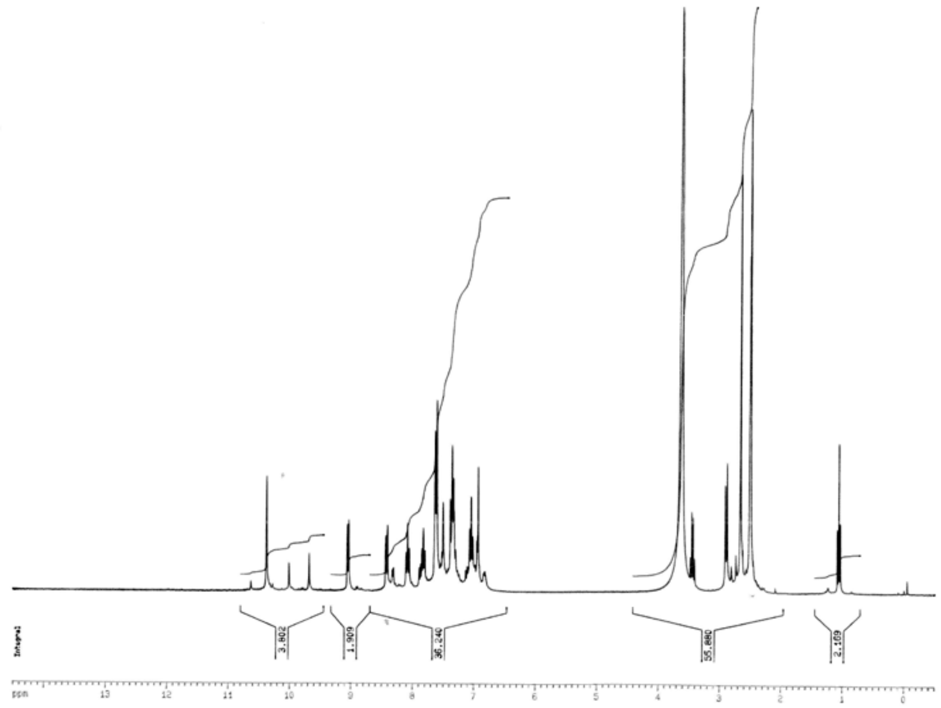

[Pd(4PLO)Cl (**5**)

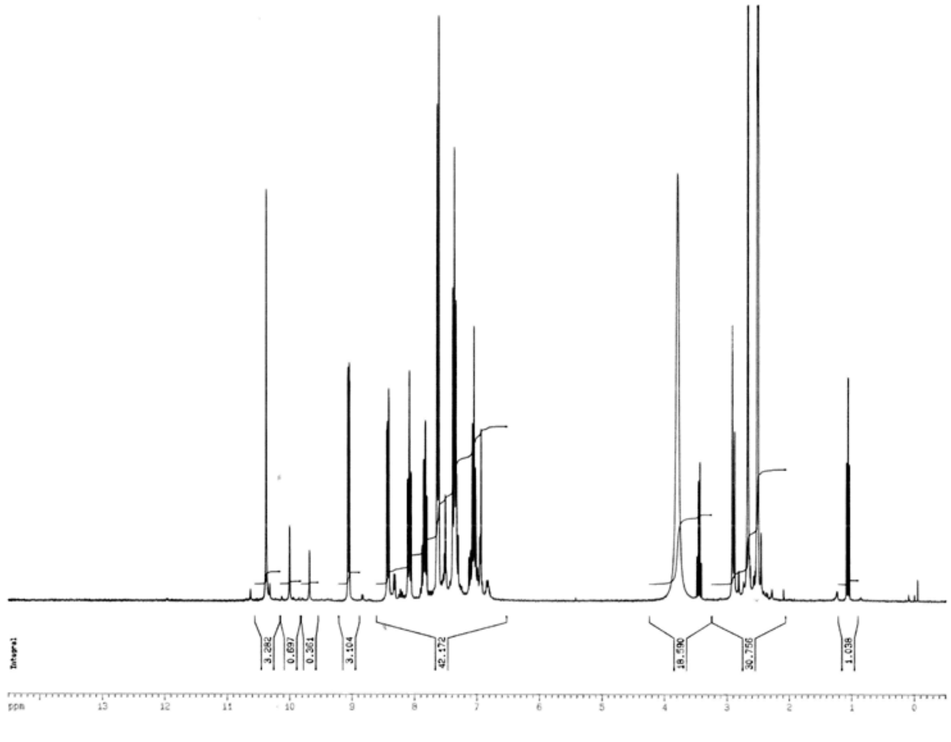

*[Pt(4PLO)Cl] (**6**)*

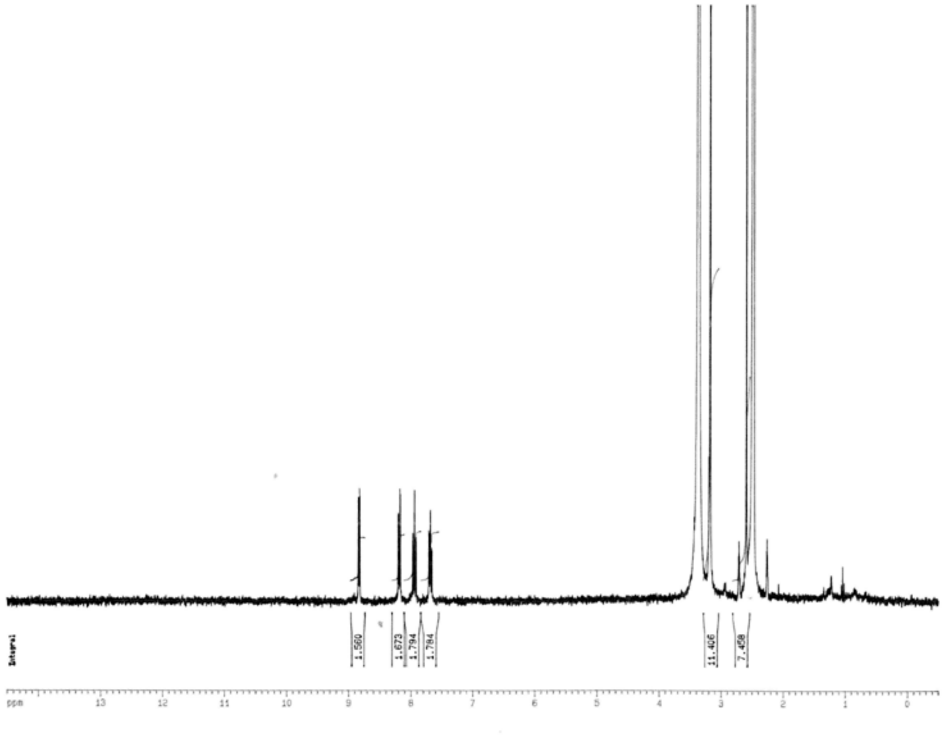

[Pd(4DMLO)Cl] (**7**)

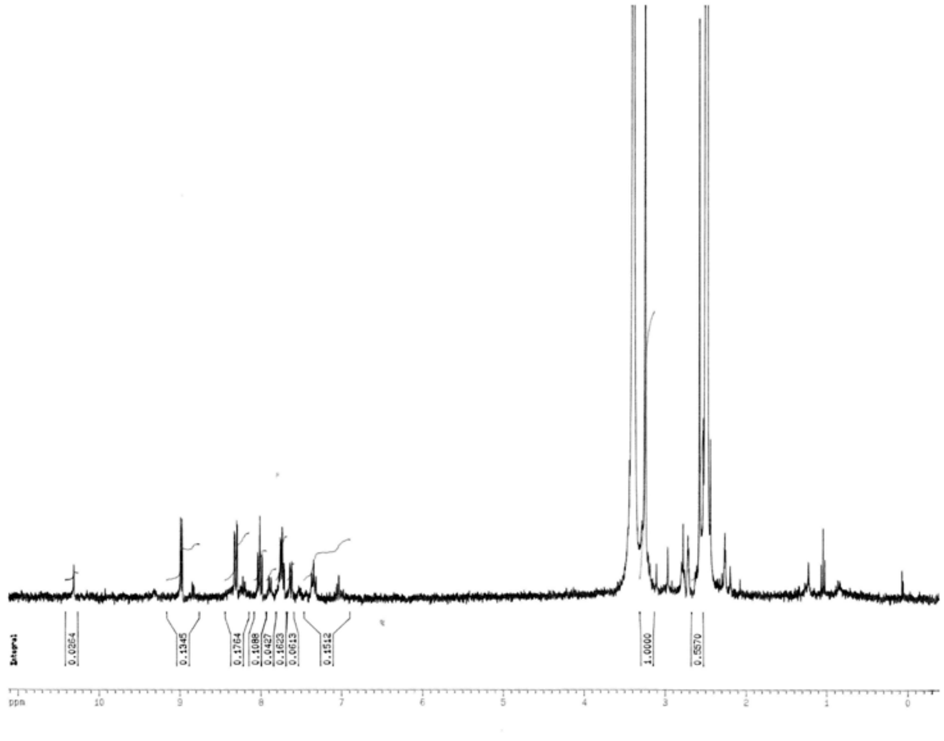

[Pt(4DMLO)Cl] (**8**)

Figure S6. ^{13}C NMR spectra

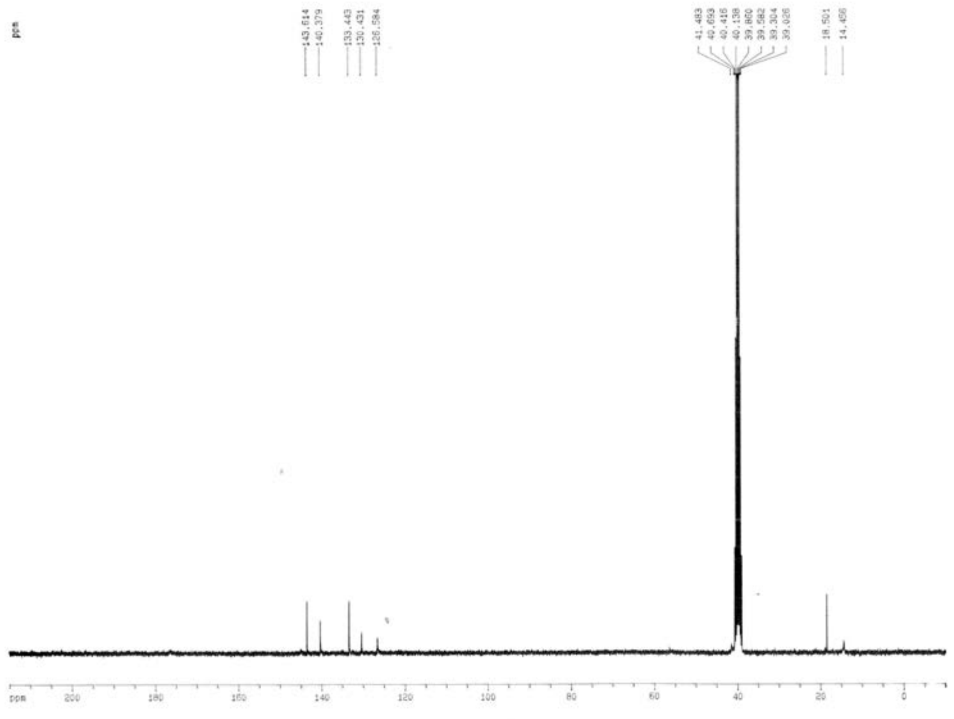

[Pd(4ELO)Cl] (**3**)

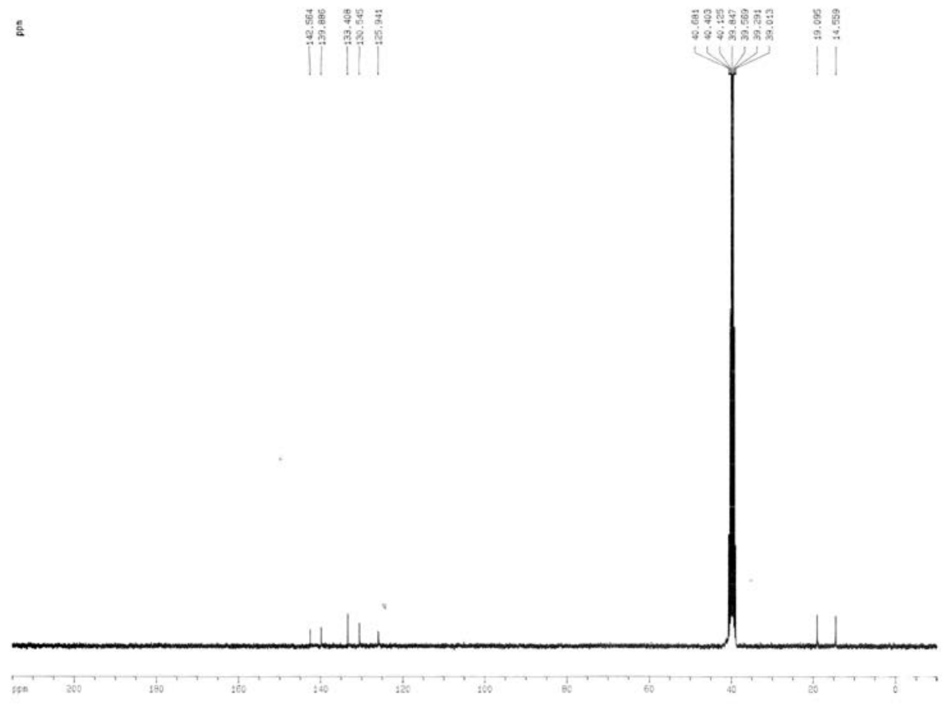

[Pt(4ELO)Cl] (**4**)

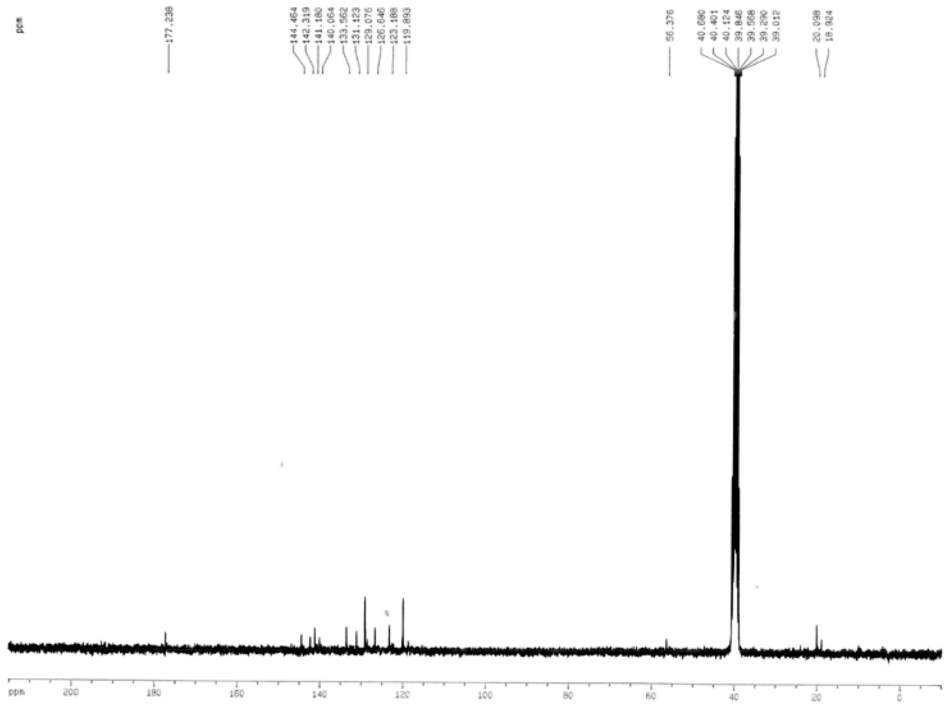

[Pd(4PLO)Cl (**5**)

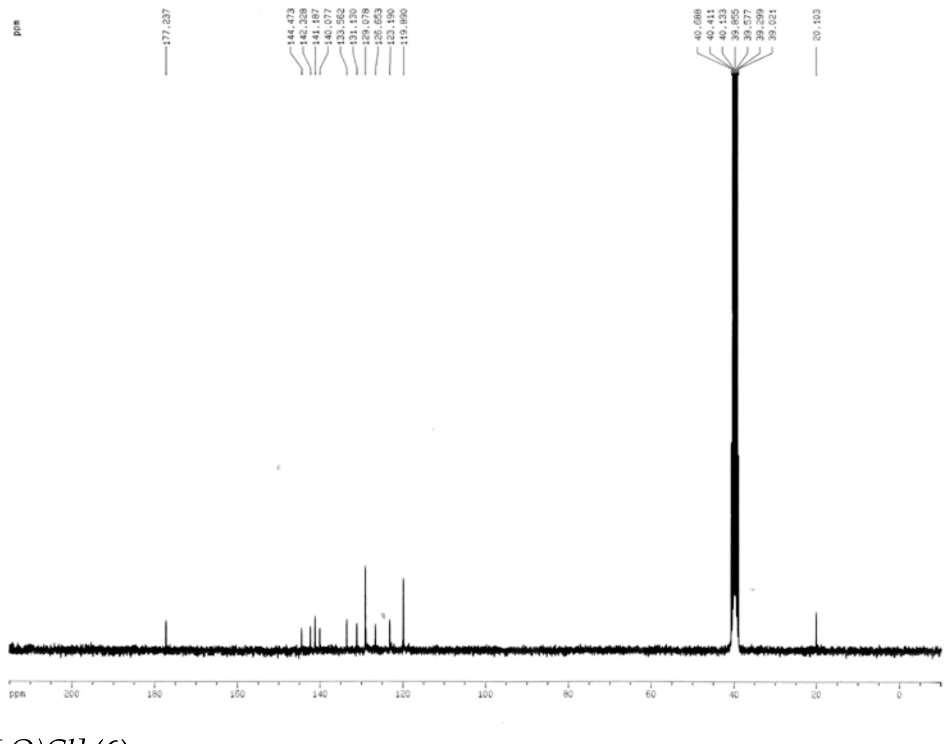

[Pt(4PLO)Cl] (**6**)

Figure S7. ^{195}Pt NMR spectra

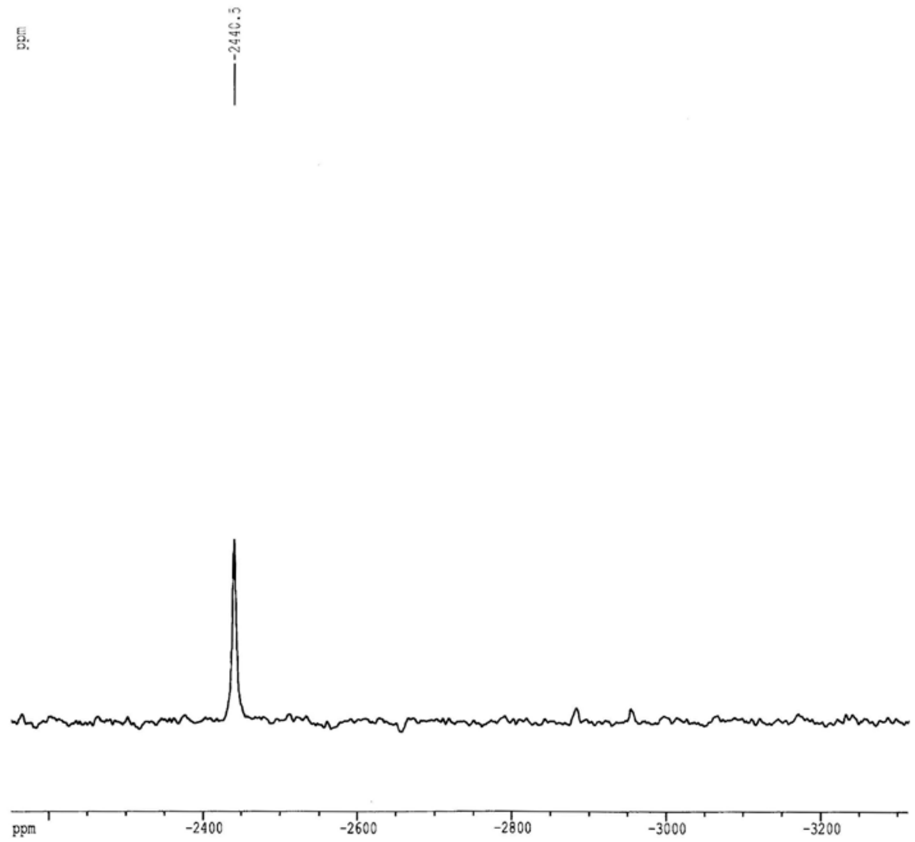

[Pt(4PLO)Cl] (**6**)

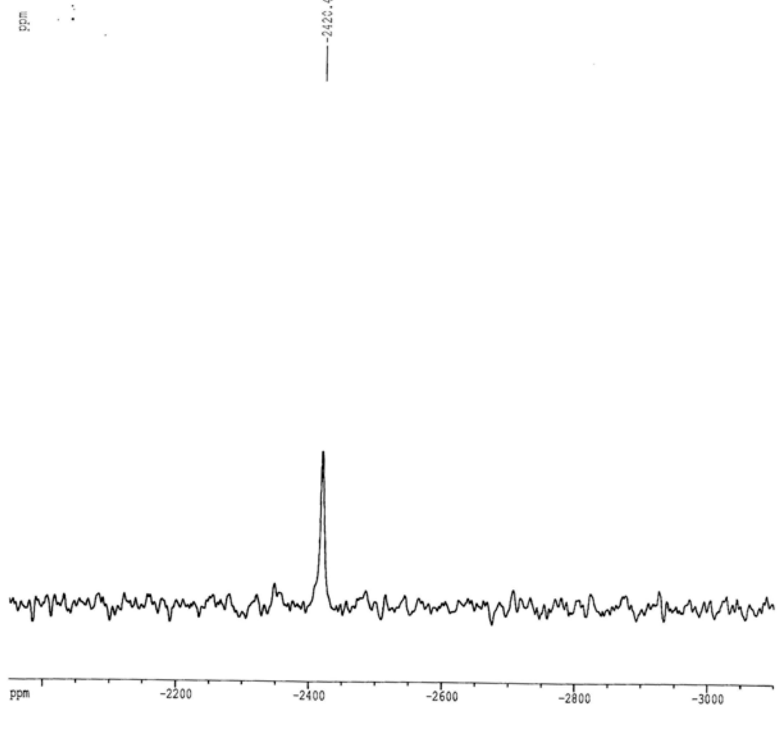

[Pt(4DMLO)Cl] (**8**)
